# Sutureless Fixation of Amniotic Membrane for Therapy of Ocular Surface Disorders

**DOI:** 10.1371/journal.pone.0125035

**Published:** 2015-05-08

**Authors:** Ilya Kotomin, Monika Valtink, Kai Hofmann, Annika Frenzel, Henning Morawietz, Carsten Werner, Richard H. W. Funk, Katrin Engelmann

**Affiliations:** 1 Department of Ophthalmology, Klinikum Chemnitz gGmbH, Chemnitz, Germany; 2 Institute of Anatomy, Medical Faculty Carl Gustav Carus, TU Dresden, Dresden, Germany; 3 Deutsche Gesellschaft für Gewebetransplantation, DGFG, Hannover, Germany; 4 Division of Vascular Endothelium and Microcirculation, Department of Medicine III, TU Dresden, Dresden, Germany; 5 CRTD / DFG-Center for Regenerative Therapies Dresden—Cluster of Excellence, Dresden, Germany; 6 Leibniz-Institut für Polymerforschung Dresden e.V., Dresden, Germany; Boston University School of Medicine, UNITED STATES

## Abstract

Amniotic membrane is applied to the diseased ocular surface to stimulate wound healing and tissue repair, because it releases supportive growth factors and cytokines. These effects fade within about a week after application, necessitating repeated application. Generally, amniotic membrane is fixed with sutures to the ocular surface, but surgical intervention at the inflamed or diseased site can be detrimental. Therefore, we have developed a system for the mounting of amniotic membrane between two rings for application to a diseased ocular surface without surgical intervention (sutureless amniotic membrane transplantation). With this system, AmnioClip, amniotic membrane can be applied like a large contact lens. First prototypes were tested in an experiment on oneself for wearing comfort. The final system was tested on 7 patients in a pilot study. A possible influence of the ring system on the biological effects of amniotic membrane was analyzed by histochemistry and by analyzing the expression of vascular endothelial growth factor-A (VEGF-A), hepatocyte growth factor (HGF), fibroblast growth factor 2 (FGF 2) and pigment epithelium-derived factor (PEDF) from amniotic membranes before and after therapeutic application. The final product, AmnioClip, showed good tolerance and did not impair the biological effects of amniotic membrane. VEGF-A and PEDF mRNA was expressed in amniotic membrane after storage and mounting before transplantation, but was undetectable after a 7-day application period. Consequently, transplantation of amniotic membranes with AmnioClip provides a sutureless and hence improved therapeutic strategy for corneal surface disorders.

**Trial Registration:**

ClinicalTrials.gov NCT02168790

## Introduction

Amniotic membrane (AM) transplantation was first introduced in the 1940ies and was a tremendous advancement for the therapy of persisting corneal defects [1]. In the 1990s, the development of methods to preserve AM further supported this therapeutic approach. Membranes were now able to be stored for several months, allowing for scheduled surgical interventions [2]. AM is sutured onto the ocular surface or into the defect area to treat corneal ulcerations of different origin and of varying depth up to perforation [3, 4], preventing progress of the disease and the eventual need for further surgical interventions like keratoplasty, conjunctival coverage, or tarsorraphy. Recently, AM transplantation was also successfully applied as an adjuvant therapy for treating bullous and band keratopathy [5, 6].

AM is composed of extracellular matrix proteins like collagens, laminins, and fibronectin [7], and releases anti-inflammatory interleukins which suppress various pro-inflammatory factors [8, 9]. Furthermore, AM contains and releases anti-angiogenic factors [8], and mediates epithelial proliferation by various growth factors [10]. Although there is growing evidence for the beneficial effects of AM, it is still not fully understood how it supports wound healing. Presumably, AM may serve as a reservoir for beneficial factors that are released upon tissue degradation. This release would lead to a wash-out of factors after transplantation. Therefore, a weekly change of AM is recommended in order to sustain healing of epithelial defects [11]. Furthermore, AM may also act as a guide rail to enhance re-epithelialization of the ocular surface [11].

From a clinical point of view, surgical manipulation of the inflamed or diseased ocular surface implies additional trauma or wounding, and side effects such as conjunctival bleeding or scarring cannot be excluded. This renders a weekly repetition of the surgical procedure not feasible. In some cases a surgical AM coverage is not possible due to logistical reasons, e.g. in the early phase of life-threatening bullous skin diseases like Lyell syndrome or Stevens-Johnson syndrome, or in multimorbid patients. One possibility to avoid surgical manipulation is to fix AM with fibrin glue onto the diseased ocular surface [12], but in a recent case report fibrin glue application onto the diseased corneal surface was considered disadvantageous, because it may mask the underlying lesion and may interfere with drug penetration [13]. The optimum would be a completely non-surgical procedure with minimal manipulation of the diseased conjunctival or corneal tissue. Therefore, we designed a system for sutureless AM coverage in which AM is mounted between two rings (AmnioClip), which can be applied like a contact lens onto the patient eye and which allows for weekly application of new AM without surgical intervention.

During the developmental process, different prototypes were designed and tested on healthy subjects. The final prototype was successfully tested in a first pilot study of patients with corneal diseases. Additionally, the biological features of AM before and after therapeutic application were examined. A manufacturing license according to national legal obligations was obtained.

## Materials and Methods

The protocol for this trial and supporting TREND checklist are available as supporting information; see S1 Checklist, S1 Protocol, and S2 Protocol (German).

### Development and manufacture of AmnioClip

Three different prototypes consisting of an inner and an outer ring for mounting AM in between the rings were developed by computer-aided design (CAD) according to clinical requirements regarding fit and tolerance. The first two prototypes were manufactured at the workshop of the Leibniz-Institute for Polymer Research Dresden (IPF Dresden, Germany) on a computerized numerical control (CNC) lathe and were made of polycarbonate (PC) or polyether ether ketone (PEEK) with a truncated, cone-shaped form, allowing the assembled rings to rest tangentially on the sclera. The third prototype, AmnioClip, had an outer silicone ring manufactured as a pressed part under clean room conditions (KET Kunststoff- und Elasttechnik GmbH, Liegau-Augustusbad, Germany) and an inner steel ring made from bended wire (FBtec Knoche & Mork GmbH, Hagen, Germany) that was laser welded (FocusControl, Braunschweig, Germany) (design and assembly of AmnioClip by FORTECH GmbH, Dresden, Germany). In addition, a sterilizable device for facilitated mounting of AM into AmnioClip was designed (FORTECH GmbH), manufactured in single parts on a CNC lathe (Kunstofftechnik Dresden, Dresden, Germany), and assembled (FORTECH GmbH). The rings were sterilized by electron beam (25 Gy; Synergyhealth, Radeberg, Germany) according to a validated procedure (KET Kunststoff und Elasttechnik GmbH) in heat sealed FLEXOPEEL sterilization bags (Kobusch-Sengewald, Warburg, Germany; Steripack 85 heat sealer, Münchner Medizin Mechanik, Munich, Germany) under clean room conditions.

### Testing on healthy subjects

During development of the ring system, the various prototypes were tested on healthy subjects (IK, KE) to examine wearing comfort. The clipped rings without AM were applied to the ocular surface after local anesthesia (0.5% proxymetacaine eye drops) under slit lamp control. Rings were left on the ocular surface for 1–2 hours. Wearing comfort was estimated in dependence on foreign body sensation and induction of pain.

### Patients and ethics statement

We conducted a monocentric pilot study at our clinic with a seven day-wearing period of AmnioClip to prove safety, wearability and fit of AmnioClip as a prerequisite to obtain a regulatory approval. From February 2011 to August 2013, seven patients were included and followed-up in the pilot study (expanded access) (see Table 1). One patient (no. 5) received treatment on both eyes due to a bilateral affection, so that altogether 8 eyes of 7 patients were treated. Four of these 7 patients received a repeated treatment: Patients no. 1, no. 3, and no. 4 received AM treatment twice, and patient no. 5 received 5 AMs (3 applications on the right eye and 2 applications on the left eye), summed up 14 AM applications altogether. All patients gave informed consent to participate in the study in written form. Consent was also given to use the data obtained in this study in electronic and in written form. Patients were anonymized according to an enrolment log. Detail has been removed from case descriptions to ensure anonymity. According to national laws it is stipulated to inform the respective ethics committee, but it was not necessary to register the study in an official registry or to obtain an ethics committee vote, because it was an expanded access study (“Heilversuch”). Despite this, we prospectively obtained a vote of the ethics committee. Study design and patient information form were approved by the local ethics committee (ethics committee of the regional medical association; approval no. EK-BR-50/10-1, date of approval December 10th, 2010). In addition, the study was registered at www.clinicaltrials.gov (ID no. NCT02168790). The authors confirm that all ongoing and related trials for this drug/intervention are registered as required by national laws.

**Table 1:** Diagnosis and application data.

Patient	Diagnosis	Number of applications	Eye	Duration (days)	Fitting and performance of AmnioClip
1	chronic transplant erosion	2	R	4	good
			R	6	good
2	contact lens associated keratitis with erosion	1	L	6	good, adapted instantly
3	progressive corneal scarring after Herpes keratitis	2	R	7	good, adapted instantly
			R	7	good, adapted instantly
4	corneal surface disorder and Fuchs endothelial dystrophy	2	R	6	good, adapted instantly
			R	6	good, at day 6 AM slackened
5	corneal surface disorder and Fuchs endothelial dystrophy	5	R	6	good, adapted instantly
			L	7	good, adapted instantly
			R	4	good, adapted instantly, ring fell out after 4 days
			L	4	good, adapted instantly, at day 4 AM slackened
			R	7	good, adapted instantly
6	chronic erosion, beginning ulceration	1	R	7	good
7	corneal ulcer	1	R	14	good, adapted instantly

### Assembly of AmnioClip and AM, placement on the eye and follow-up examinations

Cryopreserved AMs were obtained from the cornea and tissue bank Schwerin (HELIOS Kliniken Schwerin, Germany) in cooperation with the DGFG (Deutsche Gesellschaft fuer Gewebetransplantation gGmbH- German Association for Tissue Transplantation, non-profit organization, Hannover, Germany). AMs were thawed immediately before use, and mounted into AmnioClip using the accessory mounting device. Overlapping ends were cut off and stored in liquid nitrogen for further analyses. After local anaesthesia with 0.5% proxymetacaine eye drops, AmnioClip with AM was applied onto the ocular surface into the palpebral fissure. In some cases, a lid retractor was used to facilitate placement of the ring. Individual therapy regimes with local antibiotic, steroidal or caring/supporting eye drops were continued after insertion of AmnioClip. Patients were followed-up the next day after insertion of AmnioClip and then every second day by slit lamp examination. AMs were removed at day seven and patients were presented a final questionnaire to evaluate wearing comfort. In some cases, preservative-free sodium fluorescein (1.75 mg/ml) was applied as eye drops to the ocular surface to examine corneal erosion sizes under blue light. Findings were photodocumented using the Imaging Module IM 900 (Haag-Streit Deutschland GmbH, Wedel, Germany).

### Histochemical analysis

Ten AMs were examined by histochemical staining with hematoxylin-eosin (HE) after being worn by patients for treating chronic corneal transplant erosion (2x), contact lens associated keratitis with erosion (1x), progressive corneal scarring after Herpes keratitis (2x), and corneal endothelial dystrophy with epithelial edema (5x). Protruding ends that were cut off after mounting and stored in liquid nitrogen served as controls. Membranes were fixed in 10% formalin, embedded in paraffin and 5μm sections were cut on a rotary microtome (JUNG RM 2065 by Leica, Wetzlar, Germany). The sections were deparaffinized in descending ethanol concentrations to water and stained with Mayer´s hemalaun and eosin (VWR International GmbH, Darmstadt, Germany) according to routine histochemical protocols. Stained sections were dehydrated in ascending ethanol concentrations to xylol, and mounted in DePex (Serva Electrophoresis GmbH, Heidelberg, Germany). Sections were documented on a Nikon Optiphot-2 microscope with a Nikon DS-Fi1 camera driven by NIS-Elements software (Nikon GmbH, Duesseldorf, Germany).

### RNA isolation, reverse transcription and real-time PCR

Total RNA was isolated from AMs immediately after cryopreservation and thawing (AM), from AMs as tissue residues after mounting before transplantation (AM-transplant), and from AMs after being applied on the ocular surface for up to 7 days (AM-explant) using the RNeasy Plus Micro Kit (Qiagen, Hilden, Germany). Equal amounts of RNA were reverse transcribed using SuperScript II Reverse Transcriptase with random hexamer primers according to the manufacturer's instructions (Life Technologies, Darmstadt, Germany). Vascular endothelial growth factor-A (isoform VEGF-A165a), hepatocyte growth factor (HGF), fibroblast growth factor 2 (FGF-2) and pigment epithelium-derived factor (PEDF) mRNA expression was analyzed by real-time PCR using specific primers (Table 2), GoTaq qPCR Master Mix (Promega, Mannheim, Germany) and an iCycler (Bio-Rad, Munich, Germany) as described previously [14]. Briefly, after initial denaturation at 95°C for 8 min sequences were amplified in 40 cycles of denaturation at 95°C for 20 s, annealing at 58°C for 45 s, and extension at 72°C for 20 s. The translation elongation factor 2 (EF2) was used as reference gene for cDNA content normalization. After final extension at 72°C for 2 min, melt-curve analysis was performed to ensure a single amplified product in each reaction. Raw data were analyzed with iQ5 software (Bio-Rad, Munich, Germany), and evaluated using a mathematical model of relative expression ratio in real-time PCR under constant reference gene expression [15]. All assays were performed at least in triplicate and analyzed statistically by one-way ANOVA using SPSS, version 12.0 (SPSS, Chicago, IL, USA). Significance was accepted at p < 0.05.

**Table 2:** Primers used for gene expression analysis by real-time PCR.

Gene	Primers	Sequence, 5’-3’
VEGF-A165a	sense	cttgccttgctgctctacctc
	antisense	ggcacacaggatggcttga
HGF	sense	aacaatgcctctggttcc
	antisense	cgaaggcaaaaagctgtg
FGF2	sense	gagaagagcgaccctcac
	antisense	cagctcttagcagacattgg
PEDF	sense	cccgctggactatcacctta
	antisense	ccctcgggttttcttctagg
EF2	sense	atcctcaccgacatcaccaag
	antisense	ctgctctggacactggatctc

## Results

### Development of ring prototypes and self-experiment

**Fig 1:**
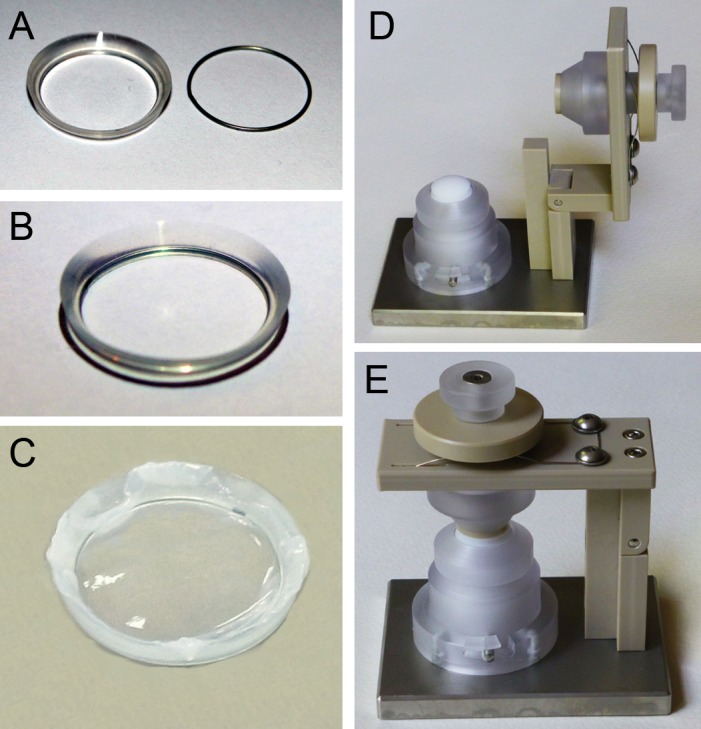
Components of AmnioClip and mounting device. A) AmnioClip, disassembled, left: outer silicone ring, right: inner steel ring; B) AmnioClip, assembled without AM, view from the inner (ocular) side; C) AmnioClip, assembled with AM, view from the inner (ocular) side, D) mounting device, open position allowing placement of the rings and AM; E) mounting device, closed “mounting” position.

AmnioClip is a dual clip ring system (Fig 1A and 1B) for sutureless application of amniotic membrane (AM) according to clinical requirements. AM is mounted between the two rings (Fig 1C) with a specially designed, accessory mounting device (Fig 1D and 1E). In initial self-experiments by two of the authors (IK, KE), the first two prototypes (not shown) caused a strong foreign body sensation with irritation of the conjunctiva already within the first hour of wearing. The stiff material was not well tolerated, and smoothening of lines and angles only slightly improved the wearing comfort. The third prototype (AmnioClip, Fig 1) was rendered suitable for clinical use. The outer silicone ring has a diameter of 23.1 mm, while the inner ring has a diameter of 18.67mm. The mounted system has a profile thickness of approx. 1.76 mm which lessens as pressure is distributed over the system during wearing, while maintaining adequate form stability. Depending on experience and with assistance, the assembly of AM into the clip ring system took approx. 5–20 minutes. During mounting, AMs were moistened with BSS. The application onto the ocular surface and insertion into the fornices then took about another 3–5 minutes. Experimental wearing by healthy subjects (IK, KE) for 1–2 hours did not induce foreign body sensation or irritation of the ocular surface due to the soft silicone material. Therefore, a small series of AmnioClip were produced and used on a small group of patients indicated for AM transplantation in a pilot study at the Department of Ophthalmology, Klinikum Chemnitz gGmbH.

### Clinical pilot study

Seven patients / eight eyes (six unilateral treatments, one bilateral treatment) were included into a clinical pilot study (expanded access study to clear safety issues) (Table 1). The average age of the patients (five women, two men) was 56.7 ± 22.5 years (24–85 years). The average duration of sutureless AM application with AmnioClip was 6.5 ± 2.4 days (4–14 days). In four cases AM application with AmnioClip was repeated: Three patients received two consecutive treatments in one eye, and one patient received three consecutive treatments in the right eye and in addition and alternating to these also received two consecutive treatments in the left eye, resulting in 14 applications altogether. Patients were informed about the indication for AM transplantation and could choose between conventional treatment (AM sutured to the conjunctiva) or sutureless application with AmnioClip as participants in the pilot study. All patients that decided for sutureless AM application gave their written consent to participate in the study after being provided detailed information. AMs were placed with the stromal side to the ocular surface, as described by Dua et al. [4]. Findings were recorded every second day and assessed with an evaluation form specifically designed for this pilot study after the intended wearing period. AM was removed after an intended wearing period of 6–7 days. In two cases AmnioClip fell out of the eye after four days without ocular injury. In another case the AM was torn in the middle at day four of wearing. One patient showed very good tolerance and retained AmnioClip for 14 days.

All patients reported that they needed to familiarize with the perception of AmnioClip at first day of wearing. Overall, patients reported a very good tolerance of AmnioClip (Table 3) and were satisfied with the effects of AM already after a few days of treatment except for one patient who did not experience subjective benefit and improvement of symptoms. Complications were not observed. Generally, all corneal indications showed improvement after treatment, and morphological complications could not be observed.

**Table 3:** Treatment tolerance; n = 14.

Subjective perception	Foreign body sensation (n)	Pain (n)
none	1	11
mild	13 (infrequently)	3 (infrequently)
moderate to considerable	0	0

The documented data were submitted by FORTECH GmbH and the DGFG to the respective authorities in order to achieve a CE certification for AmnioClip. AmnioClip is covered by patents (DE102006019017A1, EP2004115A1, US20090143792; EP 13157162.2, FORTECH GmbH), and CE certified. The CE mark, which states that the AmnioClip meets EU safety, health and environmental protection requirements, was obtained in September 2013. The tissue preparation (AM) "Humane Amnionmembran, kryokonserviert, Schwerin" (“human amniotic membrane, cryopreserved, Schwerin”), has an authorization of the national authorities (PEI.G.11579.01.1, Pharmaceutical Entrepreneur—DGFG).

### Case reports

Principally all cases could be shown, but obtaining a comparable quality of clinical photographs of patient eyes who suffer from ocular surface disorders is very difficult, because these patients have a strong protective reflex to close their eye lids and frequent nictitating. Therefore, two cases were chosen to exemplary display the fit and effect of AmnioClip on a diseased ocular surface.

#### Case 1

**Fig 2:**
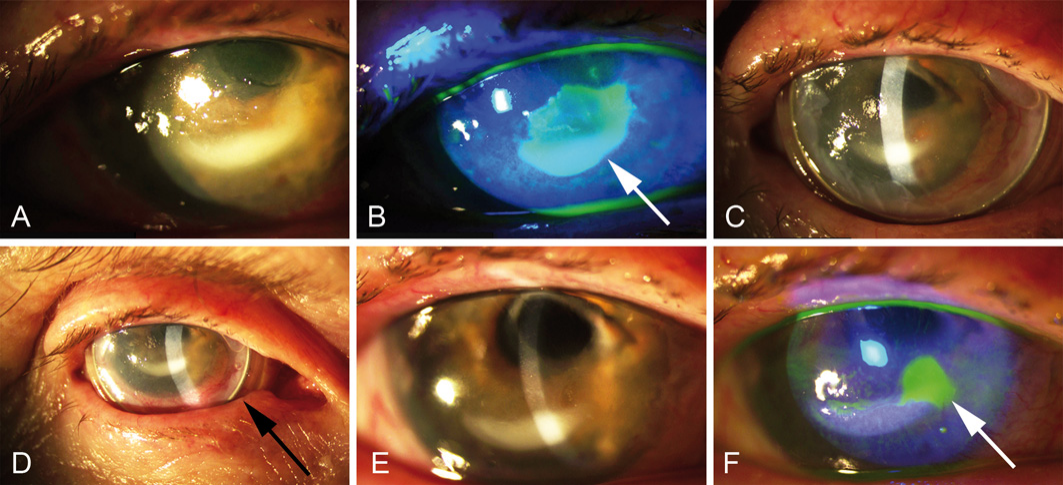
Sutureless application of AM for treating refractory epithelial erosion (case 1, patient #6). A) Slit lamp examination and B) fluorescein demonstration of the corneal erosion before sutureless AM application, arrow indicates the lesioned site; C,D) slit lamp control of AM fit at days 3 and 5 of wearing, arrow in D points on AmnioClip; E) slit lamp control and F) fluorescein demonstration of the corneal surface after removal of the AM at day 7, the arrow indicates the reduced lesion.

A 85-year old patient with unilateral pain in the right eye was diagnosed with mild conjunctival irritation and a 1x1mm epithelial erosion due to corneal decompensation after multiple retinal surgeries with silicone oil instillation. Local medication was set to dexpanthenol 50mg/ml eye drops every 2 hours during the day and ointment 50mg/g for the night and antibiotic treatment with ofloxacin 3 mg/ml eye drops every 2 hours during the day. The erosion increased in size despite local medication, and intensifying the treatment regime did not improve the symptoms. Treatment was then complemented by sutureless AM application with AmnioClip for 7 days (Fig 2), and local medication was reduced to 5x/day dexpanthenol and ofloxacin eye drops. Besides a mild sensation of pressure, no further irritations were observed by the examiner or reported by the patient. After removal of the AM the size of the erosion was distinctly reduced, so that a causal therapy to remove the silicone oil could be initiated.

#### Case 2

**Fig 3:**
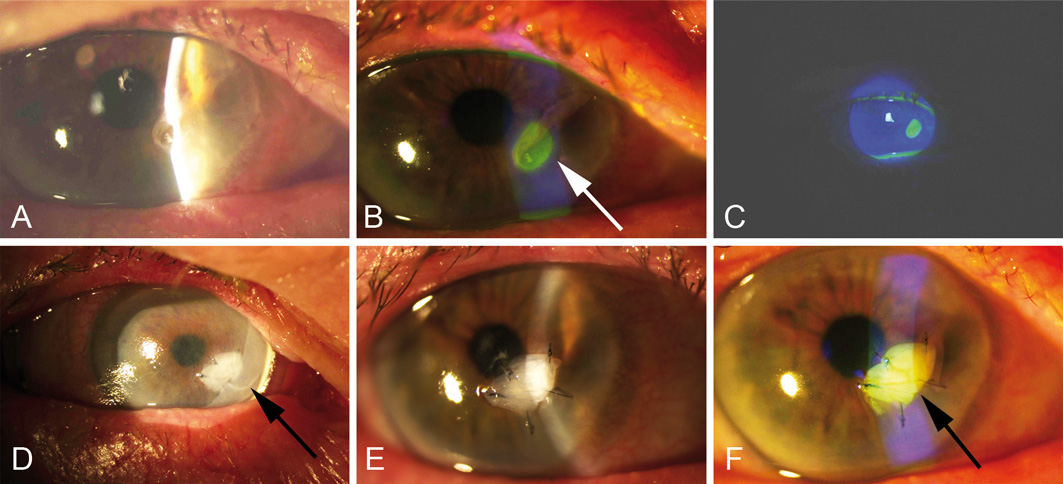
Sutureless application of AM for treating corneal ulceration and a deep stromal defect (case 2, patient #7). A) Slit lamp examination and B,C) fluorescein demonstration of the deep corneal ulceration before sutureless AM application, the arrow in B indicates the corneal ulceration site; D) slit lamp control of AM fit before removal, the arrow points on AmnioClip which was used for applying AM in sandwich technique, E) slit lamp examination and F) fluorescein demonstration of the stable AM inlay after removal of AmnioClip at day 14, the arrow indicates the AM inlay after removal of AmnioClip.

A 79-year old patient with corneal ulceration and a deep stromal defect at the 5 o´clock position was indicated for a double-layered AM transplantation. Firstly, a small AM inlay was sutured into the corneal stromal defect and the second layer was applied sutureless with AmnioClip (Fig 3), covering the entire cornea and adjacent conjunctiva. This approach was chosen to be able to exchange the second transplant within short intervals, if needed. The sutured AM patch was successfully supported by the second AM over a period of two weeks without complications. This approach was well tolerated and the AmnioClip was replaced with a bandage type contact lens during the third week.

### Histological examination of amniotic membranes after therapeutic application

**Fig 4:**
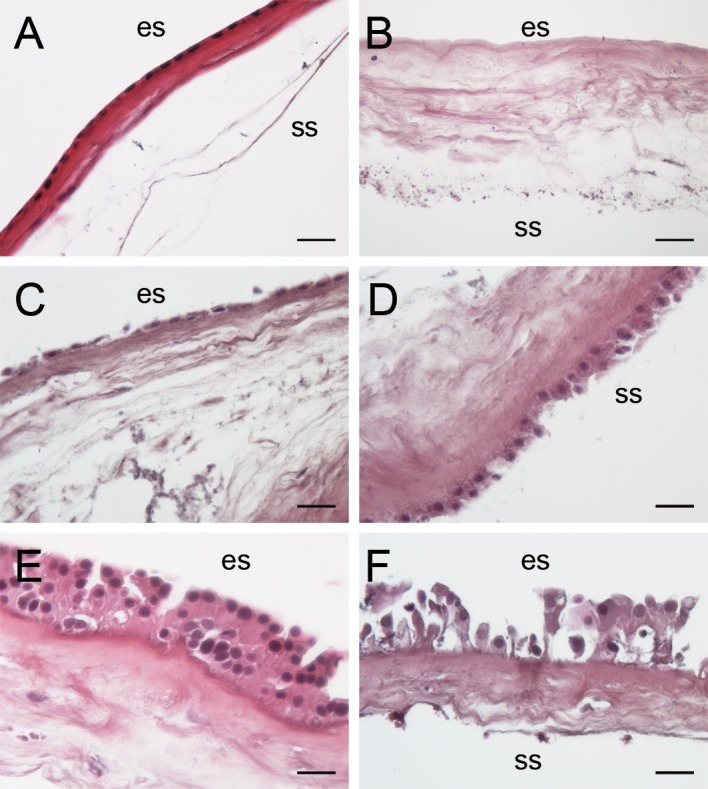
Morphology of AMs after therapeutic application. A) AM appears condensed at the site of the ring; B) AM without epithelium (patient #3 second application); C) some AMs retained their own monolayered epithelium (patient #5 first application L); D) same AM as in C, showing a double-layered epithelium with larger cells at the stromal side which faced the patient eye during application; E) some AMs had a multi-layered stratified epithelium with big, cuboidal cells with large round nuclei on the epithelial side which faced the air during application (patient #3 first application); F) same AM as in E, here the stratified epithelium at the epithelial side appeared bulky and the cells were of irregular size. HE staining; es: epithelial side; ss: stromal side; scale bar in A 100 μm, scale bar in B-F 50 μm.

Of the 14 AMs that were applied to the diseased ocular surface of patients, 10 were histologically analyzed. The AMs appeared intact with a loosened stroma. The parts of the AMs that were clipped into the holding device had a condensed stroma, but the amniotic epithelium still appeared normal (Fig 4A). Some AMs were devoid of amniotic epithelium (Fig 4B), while others were partially covered by a single layer of small squamous to cuboidal epithelial cells with flat to oval nuclei (amniotic epithelium; Fig 4C). On 4 of 10 membranes a multi-layered epithelium composed of prismatic and much larger cells with big round nuclei could be seen, which were presumed to be patient-derived epithelial cells, and not of amniotic origin (Fig 4D–4F).

### Expression of growth factors in AMs immediately before and after transplantation

**Fig 5:**
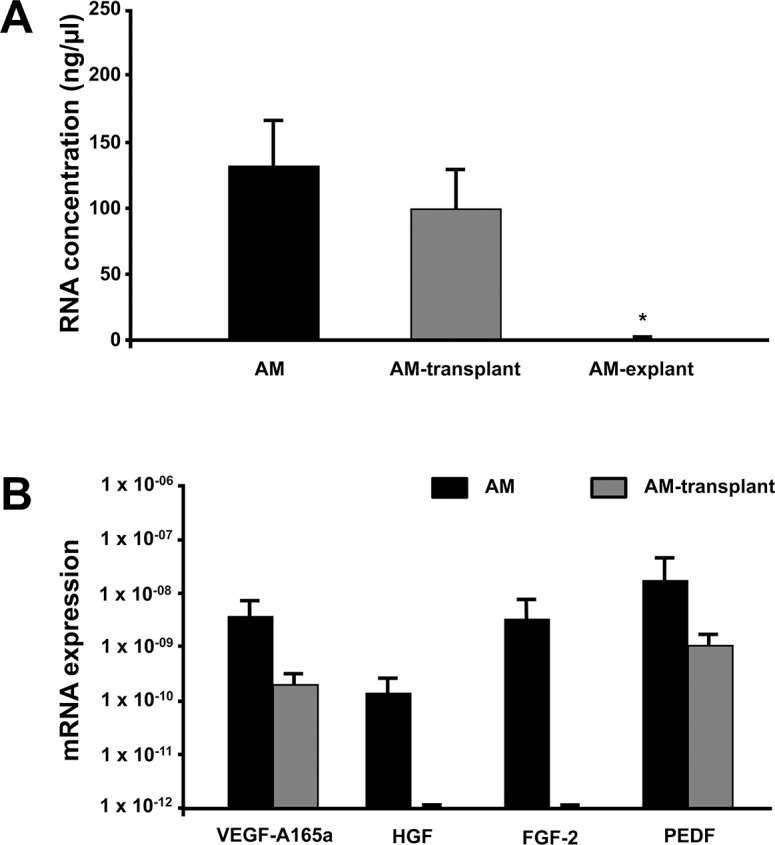
Growth factor expression in AMs at different time points during processing and use. A) Concentration of RNA from amniotic membranes directly after cryopreservation (AM), AMs after mechanical handling before transplantation (AM-transplant), or AMs after being worn on the ocular surface for one week (AM-explant) (n≥3). B) mRNA of vascular endothelial growth factor-A (isoform VEGF-A165a), hepatocyte growth factor (HGF), fibroblast growth factor 2 (FGF-2) and pigment epithelium-derived factor (PEDF) in amniotic membranes immediately after thawing (AM), or AMs after mechanical handling before transplantation (AM-transplant). Bars represent mean ± SEM of three or more measurements. *p<0.05 vs. AM.

Sufficient amounts of RNA could be isolated from stored AMs immediately after thawing (AM), or after mounting and tailoring of AMs before transplantation (AM-transplant). In contrast, after being worn for one week, successful RNA isolation could not be achieved from explanted AMs (AM-explant) (Fig 5A; n≥3). Therefore, mRNA expression of different growth factors was determined in AMs immediately after storage (AM) and after mounting and tailoring for transplantation (AM-transplant). Messenger RNA of VEGF-A, HGF, FGF-2, and PEDF was quantified in whole RNA extracts (Fig 5B). VEGF-A and PEDF mRNA could be detected in cryopreserved AMs immediately after thawing and on a lower level also in AMs after mechanical handling before transplantation, while mRNA expression of HGF and FGF-2 was only detectable in freshly thawed AM, but not after handling.

## Discussion

Multifaceted utilization of AM is aggravated by the necessity for surgical application. A sutureless application technique would have many advantages. Complications due to surgical intervention, like injury and bleeding of the conjunctiva, can be avoided. General or local anaesthesia is not necessary except for the use of anaesthetic eye drops. A sutureless application could be performed as an ambulatory treatment not only at an ophthalmological clinic, but also e.g. at intensive care units in cases of GvHD or bullous epitheliopathies like Stevens-Johnson syndrome. Expended AMs could easily be removed or replaced by new ones. Moreover, AM could be applied more often and in shorter intervals onto the patient eye, allowing for the utilization of its beneficial anti-inflammatory and wound-healing features without repeatedly imposing a surgical trauma. Handling of the membrane is also facilitated by simply mounting it into the clip ring system with a specially designed mounting device.

Publication of the development of AmnioClip and the resulting clinical data were delayed due to patenting the system. Parallel to developing AmnioClip, two similar systems were recently developed and published. ProKera was developed in the USA and is delivered as a ready-to-use product with AM mounted in a form stable PMMA ring system in a 1/1 solution of DMEM and glycerol with ciprofloxacin and amphotericin B [16, 17]. Tolerance of this system was described in a small clinical study [18]. Another ring system is comprised of a perforated titanium ring to which the AM is sutured [19]. All three systems can be used to apply AM to the ocular surface without surgical intervention. With AmnioClip the AM can be applied within a short time frame and no suturing is necessary. As a further advantage, AmnioClip can be obtained either as a ready-to-use applicable system or as single components in case users prefer to mount the AM themselves. The mounting device can be sterilized and is easy to use. This enables a repeated application of AM in a patient with individual treatment time, because all parts of the system can be stored for several months (AM up to12 months at -60°C as recommended by the DGFG). Utilization of the system without additional solutions and preservatives protects the AM from a loss of active ingredients by wash-out before application.

The silicone-based ring system aids the observed good tolerance and wearing comfort of AmnioClip. This was successfully tested in an experiment on oneself and in a pilot study on patients with diseased ocular surfaces. The DGFG plans an application for the marketing authorization of the AmnioClip plus AM at the national authorities in accordance with national statutory requirements. Furthermore, the European Commission Directive on the use and safety of cells and tissues for medical applications requires proof that the combination of amniotic membrane with a medical device is not detrimental to the AMs biological quality, which was examined before and after application of AM with AmnioClip.

Amniotic epithelial cells express and release growth factors which can protect corneal tissue against apoptosis and improve regenerative processes [10]. Recently, it was shown in a mouse model of wound healing on skin that AM exerts an anti-angiogenic effect at its epithelial side and an angiogenic effect at its stromal side [20]. As mentioned by the authors, the reports about the effects of AM on stimulation or inhibition of angiogenesis differ in the literature. Until now the decision as to whether AM is placed with its epithelial or stromal side in contact to the ocular surface is left to the surgeon [21]. Here we give evidence that the additional expression of VEGF-A, HGF, FGF-2 and PEDF. PEDF is an effective neurogenic and neuroprotective agent, but also a potent inhibitor of neovascularization [22], and likely accounts for the anti-angiogenic effect of AM. Therefore, PEDF released from transplanted amniotic membranes might mediate beneficial effects in the cornea as well, prohibiting vessel ingrowth into the wounded corneal tissue. Though VEGF-A and FGF-2 promote angiogenesis and vessel growth [23, 24], which is undesired in corneal tissue, they can act as survival factors for corneal and neural cells as well [25, 26]. Transient expression of VEGF-A might even be beneficial by promoting angiogenesis, vessel growth and survival of damaged cells especially in limbal regions due to improved supply with nutrients and oxygen to support the limbal epithelial stem cell niche [27]. However, VEGF-A expression is partially reduced in AM after freezing/thawing and AM is depleted of all factors during a 7-day treatment. HGF contributes to corneal wound healing by stimulating the proliferation of human corneal epithelial and endothelial cells [28]. Even while the HGF and FGF-2 mRNA were detected in amniotic membranes directly after thawing, but not after mounting the membranes, preformed peptides deposited in the membrane stroma might be released from transplanted AM. This could initiate regenerative processes in the state of corneal diseases. Since we could not detect a considerable amount of mRNA in explanted AMs one week after transplantation, substantial growth of amniotic cells or invading corneal cells on the transplanted AMs can be excluded.

In conclusion, the transplantation of AM using the novel, sutureless AmnioClip system might provide an improved therapeutic strategy in corneal diseases.

## Supporting Information

S1 Checklist (PDF)

S1 Protocol (PDF)

S2 Protocol (German). (PDF)
