# Supplementary material for: Sutureless Fixation of Amniotic Membrane for Therapy of Ocular Surface Disorders
Source: PLoS One. 2015 May 8;10(5):e0125035. doi: 10.1371/journal.pone.0125035 (PMC4425509; doi:10.1371/journal.pone.0125035)
Supplement: S1 Protocol — (PDF) [file pone.0125035.s002.pdf]

Ethics committee

Application
to the assessment of ethical and legal questions
of a medical research project on humans

I. General information

1. Title of the research project

Pilot study (expanded access) to evaluate the compatibility of a ring covered with an amniotic membrane (AmnioClip) for placing on the ocular surface

2. Name and address of the

a) responsible head of the clinical trial
Prof. Dr. Katrin Engelmann
Department of Ophthalmology of the Klinikum Chemnitz gGmbH, Flemmingstr. 2, 09116 Chemnitz, Germany

b) responsible project manager
Ilya Kotomin, Klinikum Chemnitz gGmbH, Flemmingstr. 2, 09116 Chemnitz, Germany

c) Investigating physician
see 2b)

3. Hospital, institute or surgery in which the research project shall be carried out

Department of Ophthalmology of the Klinikum Chemnitz gGmbH, Flemmingstr. 2, 09116 Chemnitz

4. Name and address of the managing director of the institute

Dr. Ute Dornheim (temporarily) commercial management of the Klinikum Chemnitz gGmbH
Prof. Dr. J. Klingelhöfer, medical managing director of the Klinikum Chemnitz gGmbH

5. Financing

[X] with sponsor [ ] without sponsor

Name of the sponsors

The manufacturer of the holder and clamping device is FORTECH UG (with limited liability), Heideflügel 3, 01324 Dresden, Germany

6. Was an application with the same content already been filed in another ethics committee? And if so, which? (The vote of the other ethics committee is attached.)

Yes, ethics committee of the university hospital Halle, Germany

II. Research project

1. Brief summary of the project

Surface defects of the eye are difficult to treat and occur after injuries such as acid burns, or are caused by autoimmune diseases such as rheumatoid arthritis, in which the cornea with ulceration is involved particularly at higher age. Even after penetrating keratoplasty, disturbances of the epithelization can occasionally occur. The use of commercially available eye drops to foster wound healing is generally not very effective, because they cannot support proliferation of the corneal epithelium. The treatment is usually tedious. Clinical trials with growth factor containing eye drops were carried out many years ago, but did not provide any results. Todate, locally applicable drugs based on the basis of a growth factor are not available. Thus, disturbances in wound healing require consuming and tedious ointment and drop therapies. This also involves long stays at hospi-tals for these patients. During the treatment, cloudiness often occurs in the area of the corneal stroma. Thus a visual worsening or even blindness in the affected eye may be a consequence.

In the clinical practice, surgical application of an amniotic membrane by suturing onto the ocular surface has been proven as a very good alternative. The amniotic membrane is obtained after cesarean birth (cesarean section) and can be stored for a certain time in special media or freeze-dried. Meanwhile, there are many trials on the effectiveness of this method (see accompanying review article). The amniotic membrane is sutured onto the ocular surface of the patient, i.e. the membrane is fixed with a continuous thread or with individual sutures. Amniotic membrane is considered to be poorly immunogenic and it is obviously rich in growth factors, so that the healing process strongly is promoted under normal circumstances. The clinical results are significantly better in comparison to a

conventional ointment or drop therapy or in comparison to the application of contact lenses.

The disadvantage of the surgical method is that amniotic membranes often need to be re-attached with sutures (more than once) because they can detach from the ocular surface. In addition, the threads often cause pain to the patients, so that contact lenses have to be put on in addition. The re-suturing of a membrane also means another surgical intervention on the conjunctiva, which should be impaired as little as possible. In addition, a regional block anesthesia or general anesthesia has to be performed.

The pilot study to test an amniotic holder (AmnioClip) means an optimization of the therapy, since a ring system in which the amniotic membrane is clamped and which can be placed onto the ocular surface without sutures has several advantages. First, it facilitates the handling of the membrane for the surgeon. Secons, the ring does not need to be sutured, but is worn similar to a large contact lens, and thus saves the affected persons the expensive suturing and the accompanying damage to the conjunctiva. The positioning of the membrane requires only a drop anesthesia, and the AmnioClip is not supposed to cause postoperative pain, because it is known that large contact lenses or so-called Illig-shells are well tolerated by the eye.

The pilot study is based on these abovementioned points, namely the easier handling of the amniotic membrane, the greater comfort for the patients and the possibility of an easy and rapid exchange of the membrane, if required e.g. in the course of a chronic disease.

In comparison to the standard therapy, suturing of the membrane to the diseased ocular surface, the new procedure of treatment has the advantage that the membrane is placed on the ocular surface by means of the holder, and no invasive surgical suturing techniques are necessary. This significantly simplified treatment can take place outside an operating room. An exchange of the membrane is rapidly done and can occur more frequently if required. This potentially improves the success.

The proposed project is acceptable from an ethical point of view, since it is already known that large contact lenses or scleral shells are compatible on the eye surface. The insetion of the amniotic membrane holder AmnioClip into the palpebral fissure can be controlled by means of a slit-lamp examination. The removal of the amniotic holder AmnioClip is possible at any time without problems. This is where the clinical relevance of the pilot study comes to the fore. A ring covered with an amnion which is placed as a contact lens on the eye may be inserted or removed at any time without any surgical effort.

Together with the spanned amniotic membrane, the amniotic membrane holder can be distributed by eye banks or companies to hospitals or surgeries.

The observation of a placebo group is not necessary, because suturing of an amniotic membrane has been enforced through many trials and observations against an ointment therapy.

Aim of the project:

The primary objective of this project is to examine the mounting and compatibility of the amniotic membrane holder including the amniotic membrane (AmnioClip) in the eye of the patient.
The secondary objective of the study is to examine to what extent the underlying disease can be improved during the observation period (1 week). This is done without a control group.

2. Type of the research project*

therapeutic trial	[x]	therapy	[ ]
prophylaxis	[ ]	human experimentation	[ ]
diagnostics	[ ]	miscellaneous	[ ]

3. Is it a drug testing?*

yes [ ] no [x];

if so, for which phase of the drug testing is the drug to be tested approved,

a) for the indication to be tested [ ] yes [ ] no

b) for another indication [ ] yes [ ] no if so, for which?

4. Is it a research project, to which

a) the Medicines Act [ ]

b) the Radiation Protection Regulation [ ]

c) the X-Ray Regulation [ ]

d) the Medical Devices Regulation [ ]

e) the Medical Product Law [x]

can be applied?*

On item a) Thereby, attention is to be paid to § 67 of the Medicines Act. (Notification obligation to the regional council)

In the case of radiological and / or nuclear medicine investigations only caused by the research project, we ask you to ensure that an authorization in accordance with the Radiation Protection Act by the relevant regional council has to be available.

5. Type of the research project*:

open	[x]	randomized	[ ]
blind	[ ]	multicenter	[ ]
doubleblind	[ ]	case study	[ ]
comparative	[ ]	pilot study	[ ]
		national / multinational	[ ]

6. Procedure of the clinical trial see III

III. Questions to patients and probands

10 persons are involved in the pilot study.

It is planned to involve the patients starting Feb 2011.

The postoperative observation period amounts one week. Dependent upon compatibility, the postoperative observation period may be extended to 4 weeks.

Inclusion criteria

- male and female patients between 18 and 85 years
- epithelial defects of the cornea of different genesis (eg after injury, after acid burns, in autoimmune diseases such as rheumatoid arthritis)
- incipient corneal ulcer, unless there is no bacterial infection
- existence of the declaration of patient consent

Exclusion criteria

- visible small palpebral fissure
- inflammatory bacterial diseases a causal factor for epithelial defects
- participation of the patient in another clinical trial within the last 4 weeks prior to the inclusion
- addiction or other diseases which do not allow the person concerned to evaluate the nature, scope and possible consequences of the therapeutic trial
- women who are either pregnant or breast feeding
- women of child-bearing age, except women who meet the following criteria:
    - post-menopausal (12 months natural amenorrhoe or 6 months amenorrhoe with serum FSH > 40 mlU/ml)
    - post-operative (6 weeks after bilateral ovarectomy with or without hysterectomy)
    - regular and correct application of a contraceptive method with an error rate < 7 % per year (eg implants, depot syringes, oral contraceptiva, intrauterine device – IUD). It should be considered that the combined oral contraception – as opposed to purely progesterone preparations – has a failure rate of < 1 %. Hormone coils with a Pearl index < 1 % are safer than copper coils.
    - sexual abstinence
    - vasectomy of the partner
- evidence that the patient presumably does not comply with the trial plan (e.g. lack of cooperation)

Procedure of the clinical trial

Visit 1 (Screening Visit, Baseline)

Each participating person has to be fully informed about the therapeutic trial before agreeing to participate in the therapeutic trial. The medical information and education is given personally by the investigating physician as well as in writing by the patient information sheet. Only subsequently to the clarification of all questions, the patient is asked to sign and to personally date two copies of the consent form. Afterwards, one copy of the patient information sheet / consent form is issued to the patient; the second copy shall be preserved in a document file at the trial centre.

The demographic data, anamnesis, and secondary diagnoses are acquired at the visit 1. In addition, an ophthalmological examination is carried out, and vital parameters as well as the body weight are

recorded. Among women of childbearing age, a pregnancy test will be performed. The patient will be enrolled in the therapeutic trial according to the results of verification of the inclusion exclusion criteria.

Visit 2 (OP-day)

Application of the amniotic membrane holder AmnioClip on the ocular surface.

Visit 3 and 4 (day 1 and 3 postoperative)

Verification of the position of the amniotic membrane holder AmnioClip, inspection of the eye, questions concerning pain and subjective well being. Photodocumentation.

Visit 5 (day 7 postoperative, final visit)

After one week, the amniotic membrane holder is removed and a final ophthalmological examination is performed. In addition, the subjective perception is recorded. In the strict sense, the trial is completed at this time.

Following visits (day 8 to 30)

Depending on the individual assessment, the amniotic membrane holder may be left on the cornea for more than 1 week. In this case, a final visit will be performed at a later time, but at least after 4 weeks. At this visit, the holder is removed permanently, and there is a clinical slit lamp examination.

Assessment of the effectiveness

The assessment of effectiveness is performed by daily visit of the probands and by assessment of the anterior eye segment. The primary key performance target of the trial refers to the compatibility of the developed amniotic membrane holder.

If the following points are applicable for one week:

- complete eyelid closure is possible,
- ring does not touch the cornea,
- amniotic membrane is clamped firmly in the holder and is located on the cornea,
- no pain or discomfort occurs,

then this will be considered as a success.

Secondary target figure

The secondary target figure is aimed towards the support of wound healing by the applied amniotic membrane clamped into AmnioClip. Provided that the amniotic membrane holder is well tolerated, it will be assessed whether an improvement has occurred or not at the end of the observation period.

IV. Questions about the requirements of the planned research project

1. Previous knowledge and experiences from the formulation of the question

Concerning to this, please see above under 'Introduction' (state of knowledge)

a) from preclinical trials (animal experiments, § 40 paragraph 1 section 5 AMG)
omitted

Planned test dose in humans:
Omitted
Description of the amniotic membrane holder (AmnioClip) see attachment

Specialized information or investigator brochure (for non-approved drugs) is enclosed.

Risk-benefit assessment

Due to a good compatibility, the amniotic membrane does not provides any significant side effects, and the membrane is approved for medical use. Any potential side effect can only be of mechanical nature, such as the development of dents or pressure marks in the area of the con-junctiva. The investigation of these undesirable effects is - among other things - subject of this study.
The standard procedure is to suture the amniotic membranes onto the ocular surface, so that a definite and good standard is specified in this connection. The here described new method is merely a new form of application of an amniotic membrane.

2. Information/Education

The education and patient information sheet is attached

V. Medical care

The medical care of the patients is done by Prof. Dr. Katrin Engelmann and Mr. Ilya Kotomin from the Department of Ophthalmology of the Klinikum Chemnitz gGmbH.

VI. Interim analysis and criteria for discontinuation

The therapeutic trial will be finished prematurely, if:

- two of the first five patients do not tolerate the amniotic holder,
- the rate of patient recruitment is inadequate,
- serious, unresolvable problems concerning the quality of the captured data occur,
- unforeseen circumstances occur which do not allow a continuation of the therapeutic trial,
- unacceptable risks and toxicities occur (decision in accordance with a performed new benefit-risk assessment),
- new scientific results during the term of the therapeutic trial do not permit to continue with this therapeutic trial.

The LKP decides on the discontinuation of the clinical trial.

VII. Take-over declaration for the fee (if desired)

According to the provisions of the Medicines Act (§ 40 Section 1 No. 4 AMG) and other regulations, the clinical trial of drugs in humans may only be performed, if and as long as it is directed by a physician who has at least a two-year experience in clinical testing of drugs.

Prof. Dr. Katrin Engelmann has more than two years of experience in the execution of clinial trials. Mr. Ilya Kotomin is investigating physician in the Department of Ophthalmology of the Klinikum Chemnitz gGmbH, and also has more than two years of experience in the execution of studies.
