# Supplementary material for: Sutureless Fixation of Amniotic Membrane for Therapy of Ocular Surface Disorders
Source: PLoS One. 2015 May 8;10(5):e0125035. doi: 10.1371/journal.pone.0125035 (PMC4425509; doi:10.1371/journal.pone.0125035)
Supplement: S2 Protocol — (PDF) [file pone.0125035.s003.pdf]

Ethikkommission

Antrag

zur Beurteilung ethischer und rechtlicher Fragen

eines medizinischen Forschungsvorhabens am Menschen

I. Allgemeine Angaben

1. Titel des Forschungsvorhabens

Pilotstudie zur Erprobung der Verträglichkeit eines mit Amnionmembran bespannten Ringes (AmnioClip) zum Aufsetzen auf die Augenoberfläche

2. Name und Anschrift des

a) verantwortlichen Leiters der klinischen Prüfung
Prof. Dr. Katrin Engelmann
Klinik für Augenheilkunde, Klinikum Chemnitz gGmbH, Flemmingstr. 2, 09116 Chemnitz

b) verantwortlicher Projektleiter
Ilya Kotomin, Klinikum Chemnitz gGmbH, Flemmingstr. 2, 09116 Chemnitz

c) Prüfarztes
Siehe 2b)

3. Krankenhaus, Institut oder Praxis, in dem oder in der das Forschungsvorhaben durchgeführt werden soll

Klinik für Augenheilkunde des Klinikum Chemnitz gGmbH, Flemmingstr. 2, 09116 Chemnitz

4. Name und Anschrift des geschäftsführenden Leiters der Institution

Dr. Ute Dornheim (komissarisch) Kaufmännische Geschäftsführung des Klinik Chemnitz gGmbH
Prof. Dr. J. Klingelhöfer, Medizinischer Geschäftsführer des Klinikum Chemnitz gGmbH

5. Finanzierung

[X] mit Sponsor [ ] ohne Sponsor

Name des Sponsors

Hersteller des Halters und der Aufspannvorrichtung ist die FORTECH UG (haftungsbeschränkt), Heideflügel 3, 01324 Dresden.

6. Wurde schon ein Antrag gleichen Inhalts bei einer anderen Ethikkommission gestellt? Wenn ja, bei welcher? (Votum der anderen Ethikkommission ist beigefügt.)

Ja, Ethikkommission des Universitätsklinikums Halle

II. Forschungsvorhaben

1. Kurzer Abriss des Projektes

Oberflächenstörungen des Auges sind schwer therapierbar und treten auf nach Verletzungen, z. B. Verätzungen, oder aber sind bedingt durch Autoimmunerkrankungen wie die rheumatische Arthritis, bei der es besonders im höheren Alter zur Mitbeteiligung der Hornhaut mit Ulkusbildung kommt. Auch nach der perforierenden Keratoplastik kommt es gelegentlich zu Epithelialisierungsstörungen. Die Verwendung von handelsüblichen Augentropfen zur Wundheilung ist in der Regel nicht sehr effektiv, da sie die Proliferation des Hornhautepithels nicht fördern können. Die Behandlung ist meist langwierig. Klinische Prüfungen mit wachstumsfaktorhaltigen Augentropfen wurden vor vielen Jahren durchgeführt, jedoch erbrachten sie keine Erfolge. Es gibt zurzeit kein lokal applizierbares Medikament, welches auf Wachstumsfaktorbasis beruht. Bei Wundheilungsstörungen sind daher aufwändige und langwierige Salben- und Tropftherapien notwendig, auch sind für diese Patienten immer noch lange Krankenhausaufenthalte notwendig. Häufig kommt es während der Behandlung zu Eintrübungen im Bereich des Hornhautstromas und damit zu einer Sehverschlechterung oder gar Erblindung des betroffenen Auges.

Als eine sehr gute Alternative hat sich im klinischen Alltag das Aufnähen einer Amnionmembran bewährt. Die Amnionmembran wird nach Kaiserschnittgeburten (Sectio) gewonnen und kann für eine gewisse Zeit in speziellen Medien oder auch gefriergetrocknet aufbewahrt werden. Inzwischen gibt es viele Prüfungen zur Wirksamkeit dieser Methode (siehe hierzu anliegenden Übersichtsartikel). Das Amnion wird der betroffenen Person auf die Augenoberfläche aufgenäht, d.h. die Membran wird mit einem fortlaufenden Faden oder mit einzelnen Nähten fixiert. Amnion gilt als

wenig immunogen und ist offensichtlich reichhaltig an Wachstumsfaktoren, so dass der Wundheilungsprozess in der Regel stark gefördert wird. Die klinischen Ergebnisse sind gegenüber einer konventionellen Salben- oder Tropftherapie oder aber der Applikation von Kontaktlinsen deutlich besser.

Der Nachteil der Operationsmethode beruht darin, dass Amnionmembranen häufig mehrfach aufgenäht werden müssen, da sie sich von der Augenoberfläche lösen. Zudem bereiten die Fäden den betroffenen Personen häufig Schmerzen, so dass zusätzlich Kontaktlinsen aufgesetzt werden müssen. Das Wiederaufnähen einer Membran bedeutet auch immer wieder einen chirurgischen Eingriff an der Bindehaut, die jedoch möglichst wenig mit beeinträchtigt werden sollte. Zudem muss immer eine regionale Leitungsanästhesie oder eine Vollnarkose erfolgen.

Die Pilotstudie zur Erprobung eines Amnionringes (AmnioClip) bedeutet eine Therapieoptimierung, da ein Ring, in den die Amnionmembran eingespannt wird, einerseits dem Operateur die Handhabung der Membran erleichtert, andererseits den betroffenen Personen das aufwändige Aufnähen der Membran und damit eine Schädigung der Bindehaut erspart wird. Zum Aufsetzen der Membran ist lediglich eine Tropfanästhesie notwendig. Auch ist nicht zu befürchten, dass postoperative Schmerzen auftreten, da bekannt ist, dass große Kontaktlinsen oder auch sogenannte Illing-Schalen gut am Auge toleriert werden.

Die Pilotstudie gründet sich auf diese vorgenannten Punkte, nämlich die einfachere Handhabung der Amnionmembran, den höheren Komfort für die betroffenen Personen und die Möglichkeit eines leichten und schnellen Wechselns der Membran, sollte es im Verlauf einer chronischen Erkrankung notwendig werden.

Gegenüber der Standardtherapie, nämlich dem Aufnähen der Membran, hätte das neue Behandlungsverfahren den Vorteil, dass die Membran mit Hilfe des Ringes auf die Oberfläche gelegt wird und keine invasiven chirurgischen Nahttechniken notwendig sind.

Diese deutlich vereinfachte Behandlung kann außerhalb eines OP-Saales stattfinden. Ein Austausch der Membran ist rasch und häufiger möglich, was möglicherweise den Erfolg verbessert.

Das Prüfungsvorhaben ist aus ethischer Sicht vertretbar, da bereits bekannt ist, dass große Kontaktlinsen oder Skleraschalen auf der Augenoberfläche verträglich sind. Mit Hilfe der Spaltlampenuntersuchung kann der Sitz des Amnionhalters AmnioClip kontrolliert werden und auch seine Entfernung ist jederzeit unproblematisch möglich. Gerade dies lässt die klinische Relevanz der Pilotstudie in den Vordergrund treten. Ein mit Amnion bespannter Ring, der wie eine Kontaktlinse auf das Auge gelegt wird, kann jederzeit ohne großen operativen Aufwand eingesetzt oder auch entfernt werden.

Der Amnionring AmnioClip könnte zusammen mit der aufgespannten Amnionmembran aus Hornhautbanken oder von Firmen an Kliniken oder Praxen abgegeben werden.

Die Beobachtung einer Placebogruppe ist nicht notwendig, da sich das Aufnähen von Amnionmembranen gegenüber einer Salbentherapie bereits durch viele Prüfungen und Beobachtungen durchgesetzt hat.

Ziel des Vorhabens:

Das primäre Ziel der Prüfung ist es, den Sitz und die Verträglichkeit des Amnionringes samt Amnionmembran (AmnioClip) im Auge der Patientinnen oder Patienten zu untersuchen. Das sekundäre Ziel der Prüfung ist es, zu untersuchen, inwieweit die zugrunde liegende Erkrankung, nämlich die Wundheilungsstörung, im Beobachtungszeitraum (1 Woche) gebessert werden kann. Dies erfolgt ohne Kontrollgruppe.

2. Art des Forschungsvorhabens*

Heilversuch	[x]	Therapie	[ ]
Prophylaxe	[ ]	Humanexperiment	[ ]
Diagnostik	[ ]	Sonstiges	[ ]

3. Handelt es sich um eine Arzneimittelprüfung?*

ja [ ] nein [x], wenn ja, welche Phase der Arzneimittelprüfung ist das zu prüfende Arzneimittel zugelassen,

a) für die zu prüfende Indikation [ ] ja [ ] nein

b) für eine andere Indikation [ ] ja [ ] nein wenn ja, für welche

4. Handelt es sich um ein Forschungsvorhaben, auf das

a) das Arzneimittelgesetz [ ]

b) die Strahlenschutzverordnung [ ]

c) die Röntgenverordnung [ ]

d) die Medizingeräteverordnung [ ]

e) das Medizinproduktegesetz [x]

Anwendung findet?*

zu a) Dabei ist § 67 des Arzneimittelgesetzes zu beachten. (Anzeigepflicht beim Regierungspräsidium)

Im Falle von nur durch das Forschungsvorhaben bedingten röntgenologischen und/oder nuklearmedizinischen Untersuchungen bitten wir darauf zu achten, dass eine Genehmigung nach dem Strahlenschutzgesetz durch das zuständige Regierungspräsidium vorliegen muss.

5. Typ des Forschungsvorhabens*:

offen	[x]	randomisiert	[ ]
blind	[ ]	multizentrisch	[ ]
doppelblind	[ ]	Feldstudie	[ ]
vergleichend	[ ]	Pilotstudie	[ ]
		national/multinational	[ ]

6. Prüfablauf siehe unter III

III. Fragen zu den Patienten und Probanden

Es werden 10 Personen in die Pilotstudie eingeschlossen.

Es ist geplant, die Patientinnen/Patienten beginnend mit Februar 2011 in die Pilotstudie einzubeziehen. Der Nachbeobachtungszeitraum betrifft eine Woche, kann jedoch - je nach Verträglichkeit - auf bis zu 4 Wochen ausgedehnt werden.

Einschlusskriterien

- Männliche und weibliche Patienten zwischen 18 und 85 Jahren
- Epitheldefekte der Hornhaut unterschiedlicher Genese (z.B. nach Verletzung, nach Verätzung, bei Autoimmunerkrankungen wie z.B. rheumatoide Arthritis)
- Beginnendes Hornhautulkus, soweit keine bakterielle Entzündung vorliegt
- Vorliegen der Patienteneinwilligungserklärung

Ausschlusskriterien

- sichtbar kleine Lidspalte
- entzündliche bakterielle Erkrankung als Ursache des Epitheldefekts
- Teilnahme der Patientin/des Patienten an einer anderen klinischen Prüfung innerhalb der letzten 4 Wochen vor dem Einschluss
- Sucht- oder sonstige Erkrankungen, die es der/dem Betreffenden nicht erlauben, Wesen und Tragweite sowie mögliche Folgen der klinischen Prüfung abzuschätzen
- schwangere oder stillende Frauen
- Frauen im gebärfähigen Alter, außer Frauen, die die folgenden Kriterien erfüllen:
    - Post-menopausal (12 Monate natürliche Amenorrhoe oder 6 Monate Amenorrhoe mit Serum FSH > 40 mlU/ml)
    - Postoperativ (6 Wochen nach beidseitiger Ovarektomie mit oder ohne Hysterektomie)
    - Regelmäßige und korrekte Anwendung einer Verhütungsmethode mit Fehlerquote < 7 % pro Jahr (z. B. Implantate, Depotspritzen, orale Kontrazeptiva, Intrauterinpessar – IUP). Dabei ist zu berücksichtigen, dass die kombinierte orale Kontrazeption – im Gegensatz zu reinen Progesteronpräparaten – eine Versagerquote von < 1 % hat. Hormonspiralen sind mit einem Pearl Index < 1 % sicherer als Kupferspiralen.
    - Sexuelle Enthaltsamkeit
    - Vasektomie des Partners
- Anzeichen dafür, dass die Patientin/der Patient den Prüfplan voraussichtlich nicht einhalten wird (z. B. mangelnde Kooperationsbereitschaft)

Prüfungsablauf

Visite 1 (Screening Visit, Baseline)

Jede teilnehmende Person muss vor Aufnahme in die Klinische Prüfung umfassend über dieselbe informiert werden. Die Aufklärung erfolgt persönlich durch die behandelnde Ärztin/den behandelnden Arzt und schriftlich durch die Patienteninformation. Erst nach Klärung aller Fragen wird die teilnehmende Person gebeten, zwei Exemplare der Einwilligungserklärung zu unterschreiben und eigenhändig zu datieren. Anschließend wird ihr ein Exemplar der Patienteninformation/Einwilligungserklärung ausgehändigt; das zweite Exemplar wird im Prüfzentrumsordner aufbewahrt.

Bei Visite 1 erfolgt die Erfassung der demografischen Daten, der Anamnese und der Neben-diagnosen. Außerdem erfolgen eine ophthalmologische Untersuchung sowie das Aufzeichnen der

Vitalparameter und des Körpergewichts. Bei Frauen im gebärfähigen Alter wird außerdem ein Schwangerschaftstest durchgeführt. Nach der abschließenden Prüfung der Ein- und Ausschlusskriterien erfolgt die Aufnahme der Patientin/des Patienten in die Prüfung.

Visite 2 (OP-Tag)

Aufbringen des Amnionringes AmnioClip auf die Hornhaut.

Visite 3 und 4 (Tag 1 und 3 postoperativ)

Überprüfung des Sitzes des Amnionringes AmnioClip, Inspektion des Auges, Frage nach Schmerzen und subjektivem Befinden.

Visite 5 (Tag 7 postoperativ, Abschlussvisite)

Nach einer Woche wir der Ring entfernt und es erfolgt eine ophthalmologische Untersuchung. Außerdem wird das subjektive Empfinden erfragt. Die Prüfung im engeren Sinne ist zu diesem Zeitpunkt abgeschlossen.

Folgevisiten (Tag 8 bis 30)

Je nach individueller Begutachtung kann der Ring auch länger als 1 Woche auf der Hornhaut verbleiben. In diesem Fall findet auch zu einem späteren Zeitpunkt, spätestens jedoch nach 4 Wochen nochmals eine abschließende Visite statt. Bei dieser Visite wird der Ring endgültig entfernt und es erfolgt eine klinische Untersuchung an der Spaltlampe.

Beurteilung der Wirksamkeit

Die Beurteilung der Wirksamkeit erfolgt durch die tägliche Visite der Probanden und Beurteilung des vorderen Augenabschnitts.

Die primäre Zielgröße der Prüfung bezieht sich auf die Verträglichkeit des entwickelten Amnionhalters. Sind eine Woche lang folgende Punkte zutreffend:

- vollständiger Lidschluss möglich,
- Ring sitzt nicht auf der Kornea auf,
- Amnionmembran ist fest im Ring eingespannt und liegt auf der Kornea auf,
- es treten keine Schmerzen oder Unwohlsein auf,

so wird dies als Erfolg gewertet.

Sekundäre Zielgröße

Die sekundäre Zielgröße zielt auf die Unterstützung der Wundheilung durch die aufgelagerte, in den Amnionring eingespannte Membran hin. Vorausgesetzt, der Amnionring AmnioClip wurde so lange gut toleriert, wird nach einer Woche beurteilt, ob eine Besserung eingetreten ist oder nicht.

IV. Fragen zu den Voraussetzungen des geplanten Forschungsvorhabens

1. Bisherige Erkenntnisse und Erfahrungen zur Fragestellung

Siehe hierzu bitte Einleitung (Stand des Wissens)

a) aus den vorklinischen Untersuchungen (Tierversuche, § 40 Abs. 1 Ziff. 5 AMG)
entfällt

Geplante Prüfdosis beim Menschen:
Entfällt
Beschreibung des Amnionrings siehe Anlage

Fachinformation bzw. Investigatorbroschüre (bei nicht zugelassenen Wirkstoffen) ist beigelegt.

<u>Risiko-Nutzen-Abwägung</u>

Aufgrund der guten Verträglichkeit gibt es durch die Amnionmembran keine wesentlichen Nebenwirkungen, die beschrieben sind. Da auch das Material des Amnionringes AmnioClip für Medizinprodukte zugelassen ist, sind hier keine Nebenwirkungen zu erwarten. Die Nebenwirkungen können lediglich mechanischer Art sein, z. B. die Entwicklung von Dellen oder Druckstellen im Bereich der Konjunktiva. Die Untersuchung dieser unerwünschten Auswirkungen ist u. a. Gegenstand der Pilotstudie.

Ein Standardverfahren ist das Aufnähen von Amnionmembranen, so dass hier ein definitiver und guter Standard vorgegeben wird. Bei der neuen Methode handelt es sich lediglich um eine neue Form der Aufbringung der Amnionmembran.

2. Aufklärung

Die Aufklärung und Patienteninformation ist angefügt

V. Ärztliche Betreuung

Die Betreuung der Patienten erfolgt durch Prof. Dr. Katrin Engelmann und Herrn Ilya Kotomin der Augenklinik des Klinikum Chemnitz gGmbH

VI. Zwischenauswertung und Abbruchkriterien

Die klinische Prüfung wird vorzeitig zu beenden, wenn

- zwei der ersten fünf Patientinnen/Patienten den Amnionring nicht tolerieren
- die Patientenrekrutierungsrate unzulänglich ist
- gravierende, nicht zu klärende Probleme mit der Qualität der erhobenen Daten auftreten
- unvorhersehbare Umstände im jeweiligen Prüfzentrum eingetreten sind, die eine Weiterführung der klinischen Prüfung nicht zulassen
- unvertretbare Risiken und Toxizitäten aufgetreten sind (Entscheidung nach erfolgter neuer Nutzen-Risiko-Abwägung)
- neue wissenschaftliche Erkenntnisse während der Laufzeit der Prüfung die Weiterführung derselben nicht erlauben

Über den Abbruch der Prüfung entscheidet die LKP.

VII. Übernahmeerklärung für die Gebühr (soweit gewünscht)

Nach den Bestimmungen des Arzneimittelgesetzes (§ 40 Abs. 1 Nr. 4 AMG) darf die klinische Prüfung von Arzneimitteln beim Menschen neben weiteren Vorschriften nur durchgeführt werden, wenn und solange sie von einem Arzt geleitet wird, der mindestens eine zweijährige Erfahrung in der klinischen Prüfung von Arzneimitteln nachweisen kann.

Prof. Dr. Katrin Engelmann hat eine mehr als zweijährige Erfahrung in der Durchführung von Studie. Herr Ilya Kotomin ist Prüfarzt in der Augenklinik des Klinikum Chemnitz gGmbH und besitzt ebenfalls zweijährige Erfahrungen in der Durchführung klinischer Studien.
